# Body Condition, Metabolic Parameters and Semen Traits in Purebred Spanish Horses

**DOI:** 10.3390/ani16152347

**Published:** 2026-08-01

**Authors:** Nerea Latorre, Concepción Gómez-Cuétara, Ester Cañizares, Francisco Crespo, Verónica Pérez-Aguilera, Juan Ángel Laborda-Gomariz, Ana Josefa Soler, Ana Sanchez-Rodriguez, Eduardo R. S. Roldan

**Affiliations:** 1 Department of Biodiversity and Evolutionary Biology, National Museum of Natural Science (CSIC), C/José Gutiérrez Abascal, 2, 28006 Madrid, Spain; nerea.latorre@mncn.csic.es (N.L.); ester.canizares@mncn.csic.es (E.C.); 2 Los Callejones del Duende Equine Reproduction Center, Ctra. de Colmenar a Aranjuez, 28300 Aranjuez, Madrid, Spain; gcuetara@ucm.es; 3 Military Center for Horse Breeding (CCFAA), C/Arsenio Gutiérrez Palacios s/n, 05005 Ávila, Castilla y León, Spain; fcrecas@oc.mde.es (F.C.); vperagu@gmail.com (V.P.-A.); 4 SaBiO IREC (CSIC-UCLM-JCCM), ETSIAMB, University Campus, 02071 Albacete, Spain; juanangel.laborda@uclm.es (J.Á.L.-G.); anajosefa.soler@uclm.es (A.J.S.)

**Keywords:** Purebred Spanish horses, body condition score, metabolic parameters, endocrine parameters, seminal quality

## Abstract

The growing relevance of the Purebred Spanish horse has driven the extensive use of assisted reproductive technologies to preserve elite stallion genetics. Optimal reproductive performance is intrinsically linked to systemic health and high semen quality. This study aimed to evaluate the impact of body condition on the stallion’s metabolic and endocrine profiles and their seminal parameters. Semen and blood samples were collected from 26 stallions (February–May in 2024–2025) whose body condition score (BCS) was assessed using the Henneke scale. They were classified into two groups, low BCS with a score range of 4–6 (*n* = 14) and high BCS with a score range of 7–8 (*n* = 12). The low BCS is regarded as a normal condition, whereas the high BCS is regarded as obesity. Metabolic and endocrine parameters and semen quality variables were analyzed using ANOVA, and principal component analyses (PCA). Stallions with high BCS showed significantly increased insulin and triglycerides concentrations (*p* = 0.0263 and *p* = 0.0046, respectively), with a positive correlation between BCS and insulin levels (r = 0.576, *p* = 0.04), as well as between BCS and triglyceride levels (r = 0.525, *p* = 0.0049). A significantly lower sperm count was observed in stallions with high BCS (*p* < 0.0001), together with lower total sperm motility (*p* = 0.0055), when compared to stallions with low BCS. PCA of blood parameters revealed the first component (PC1) associated with glucose, insulin, triglycerides, and cholesterol. In semen’s PCA, PC1 was associated with quality parameters, while PC2 was influenced by total sperm count. No direct correlation was found between PCA components and BCS. However, integration of both PCA showed that increased insulin, triglycerides, and cholesterol were significantly associated with a reduced total sperm count. These findings suggest that reproductive changes in stallions with high BCS are primarily mediated by metabolic and endocrine dysfunction rather than adiposity per se.

## Simple Summary

Preserving the genetics of elite Purebred Spanish horses is increasingly important, but success relies heavily on the stallions’ health and semen quality. This study investigated how a stallion’s body condition affects its metabolic health and sperm quality. By comparing stallions with a high body condition score, indicative of obesity, to those with a normal body condition, we found that stallions with a high score had significantly higher blood levels of insulin and fat (triglycerides). Crucially, stallions with a high body condition score produced lower sperm numbers with reduced movement capabilities. Further analysis revealed that such negative changes in semen quality are directly linked to altered hormone levels in the blood, rather than to the body fat itself. This research would benefit horse breeders and veterinarians as it reveals that keeping stallions with a healthy body condition is vital for their sperm traits. By managing a horse’s diet, and preventing high body condition scores, breeders can improve semen quality and better preserve valuable horse genetics.

## 1. Introduction

The Purebred Spanish Horse is one of the most prominent equine breeds in the Iberian Peninsula. It is highly valued in the livestock sector for its distinct morphology and temperament, and increasingly for its sporting performance [1]. The breed also represents a hallmark of Spanish culture and tradition [2]. Currently, equine livestock production is primarily oriented towards economic activities such as leisure and equestrian sports [3].

Equine reproduction increasingly relies on assisted reproductive technologies (ART), whose success depends on key semen parameters such as sperm motility, which, as demonstrated in other species including humans, are directly impaired by obesity [4]. Improved feed quality, combined with reduced physical activity, has led to increased obesity rates. Purebred Spanish horses are particularly prone to adiposity when caloric intake exceeds maintenance requirements [5]. Furthermore, as obese horses appear phenotypically similar to those that are merely overweight, diagnosis is frequently delayed until the onset of obesity-related pathologies [6].

The World Health Organization (WHO) defines obesity as the abnormal or excessive accumulation of fat that may adversely affect health. The Henneke scale is widely employed for routine assessments of body condition in horses [7]. This methodology involves evaluating fat deposition across various anatomical regions, including the neck, shoulders, ribs, croup, topline, and the area over the thigh. The scale ranges from 1 to 9; horses with an ideal body weight typically score between 4 and 6, whereas those scoring 7 or above are considered obese. However, adiposity patterns are not always homogeneous, and factors such as muscle development, intestinal fill, and evaluator bias can limit the reliability of body condition scoring (BCS) scales when utilized in isolation [8]. Thus, it is essential to complement physical assessments with blood analyses to characterize the endocrinological and metabolic status of the animal. Adipose tissue functions as an active endocrine organ, secreting adipokines and hormones such as leptin, adiponectin, and resistin. A pathological expansion of adipose tissue results in elevated levels of these signaling proteins, which directly influence the development of metabolic diseases [9]. A hallmark of these diseases is insulin resistance, which impairs the action of the hormone responsible for glucose uptake, lipogenesis promotion, and the inhibition of protein degradation [10]. Dysregulation of circulating insulin levels is currently regarded as the most prevalent endocrine disorder in equine medicine [11]. Regarding leptin, circulating concentrations rise in proportion to adiposity, altering carbohydrate metabolism and cardiovascular function [12]. Conversely, adiponectin—which promotes fatty acid oxidation, inhibits hepatic glucose production, and exerts anti-inflammatory effects is typically downregulated in obesity [13]. Furthermore, obesity contributes to excess circulating non-esterified fatty acids (NEFAs).

Numerous studies across diverse animal models have demonstrated that obesity is directly linked to impaired reproductive capacity. The decrease in adiponectin levels has a deleterious impact on gonadal activity [14]. In addition, fluctuations in NEFA levels can induce lipotoxicity and oxidative stress within reproductive cells, thereby impairing spermatogenesis. Additionally, while low-density lipoprotein cholesterol (LDL-C) and circulating triglycerides are known to influence male fertility, a consensus regarding their direct impact on sperm quality has yet to be established [15]. In this sense, obesity may be a primary driver of hypogonadism and can adversely affect various seminal parameters, either through direct alterations in Sertoli cell function or indirectly via increased scrotal temperature and the sequestration of lipophilic toxic substances [16]. Obesity compromises in vitro fertilization success by increasing oxidative stress, triggering cellular apoptosis, and diminishing the energy supply to the testes [17]. In humans, semen parameters have been shown to deteriorate as body mass index (BMI) increases, characterized by a reduction in total sperm count and a lower percentage of motile sperm [18]. Furthermore, clinical evidence has linked obesity to increased sperm DNA fragmentation and a reduction in mitochondrial membrane potential, which may partially explain the observed decline in sperm motility [19].

The impact of obesity on Purebred Spanish Horses reproductive capacity is of paramount importance. Currently, obesity rates within the equine species are increasing [20]. Specifically, Sánchez-Guerrero et al. [1], determined that obesity affects approximately 70% of the Purebred Spanish horse population reared in Spain. Despite this, few studies have addressed the specific impact of adiposity on stallion fertility. This gap in knowledge represents a significant concern, as obesity impairs quality of life and necessitates dietary energy restrictions for effective management [21]. Therefore, investigating Purebred Spanish stallions is essential to characterize the physiological and metabolic alterations that spermatozoa undergo in obese animals. Such insights could ultimately improve semen quality and enhance the overall efficiency of ART.

The objective of this study was to investigate Purebred Spanish Horse stallions with varying body condition scores and metabolic-endocrine profiles to evaluate the potential impact of these parameters on semen quality.

## 2. Materials and Methods

### 2.1. Animals

A total of 26 adult Purebred Spanish Horse stallions were included in the study. All animals were in good health and were housed in individual stalls with ad libitum access to water and pasture. Sampling was conducted between February and May across two consecutive breeding seasons (2024 and 2025), with a different subset of stallions sampled each season in order to increase the overall sample size. Semen samples were collected by trained personnel as part of the routine reproductive management of the stallions. For each stallion, three ejaculates were collected over three consecutive weeks.

A single blood sample was collected from each stallion on the same day as the first semen collection, coinciding with the routine clinical blood analysis performed for each animal. Since stallions were sampled across two consecutive breeding seasons, blood collection dates ranged from February 2024 to May 2025 overall. Each stallion was sampled during only one of the two breeding seasons. No additional experimental procedures were performed on the animals; therefore, no ethics committee approval was required under current Spanish and European legislation (Royal Decree 53/2013, Directive 2010/63/EU).

#### 2.1.1. Body Condition Evaluation

Body condition was assessed using the Henneke body condition scoring (BCS) [7], which evaluates adiposity in horses on a 9-point scale, where 1 indicates emaciated and 9 indicates extreme obesity. The scoring is based on visual evaluation and palpation of subcutaneous fat deposits of six anatomical regions: the neck, withers, shoulder, ribs, loin, and tailhead. Based on their BCS, stallions were classified into two groups: low BCS (4–6, *n* = 14), considered within the normal range, and high BCS (7–8, *n* = 12), considered indicative of obesity. Given that BCS assessment involves a degree of subjectivity inherent to visual and palpation-based scoring systems, stallions classified as BCS 6 or 7 may exhibit substantial phenotypic overlap. To minimize this potential source of misclassification and to more clearly isolate the effect of extreme body condition, a secondary analysis was performed comparing only stallions at the extremes of the BCS distribution (BCS 4–5, *n* = 5, vs. BCS 8, *n* = 5).

#### 2.1.2. Evaluation of Endocrinological Parameters

Blood samples were collected by direct jugular venipuncture in the morning under fasting conditions, prior to the first daily feeding. From each stallion, two blood tubes (EDTA and no additive) were drawn and centrifuged at 2000× *g* for 10 min. The EDTA-containing tube was used for plasma collection, while the other tube without additive was used for serum collection. Both tubes were stored at −20 °C until analysis. Serum concentrations of glucose, high-density lipoprotein cholesterol (HDL-C), and triglycerides were analyzed using enzymatic methods on a Beckman Coulter AU700 automated analyzer (Beckman Coulter, Brea, CA, USA) with manufacturer-supplied reagents. Specifically, glucose was determined by the hexokinase/glucose-6-phosphate dehydrogenase enzymatic method (measurement range: 10–810 mg/dL; intra-assay CV: 0.3–0.7%; total CV: 0.6–0.9%), HDL-C by a direct homogeneous selective enzymatic method without precipitation (measurement range: 7–400 mg/dL; intra-assay CV: <3%; total CV: <5%), and triglycerides by the glycerol-phosphate oxidase/peroxidase colorimetric enzymatic method (measurement range: 10–1000 mg/dL; intra-assay CV: 0.49–0.64%; total CV: 1.41–1.65%). Non-esterified fatty acid (NEFA) concentrations were also quantified on the Beckman Coulter AU700 automated analyzer using Randox NEFA reagents (Randox Laboratories, Crumlin, UK) by the acyl-CoA synthetase/acyl-CoA oxidase colorimetric enzymatic method (measurement range: 0.072–2.24 mmol/L; total CV: <5%). Insulin concentration was quantified by chemiluminescence immunoassay on the Beckman Coulter Access analyzer (Beckman Coulter, Brea, CA, USA) using the Access Ultrasensitive Insulin reagent (Beckman Coulter, Brea, CA, USA; LOD: 0.011 µIU/mL; measurement range: 0.011–300 µIU/mL; intra- and inter-assay CV: ≤6%), which was originally formulated for human insulin and therefore validated in-house for use in equine serum through parallelism studies and intra- and inter-assay precision assessment. Finally, leptin concentration was determined by equine-specific sandwich quantitative ELISA using the Horse Leptin ELISA Kit (MyBioSource, San Diego, CA, USA; sensitivity: <0.013 ng/mL; detection range: 0.04–40 ng/mL). This kit is designated for Research Use Only (RUO), as no CE-IVD validated diagnostic method is currently available for equine leptin quantification.

### 2.2. Semen Collection and Processing

All reagents used throughout the study were obtained from ThermoFisher Scientific, Inc. (Waltham, MA, USA), unless otherwise specified.

Semen samples were collected using a Missouri-style artificial vagina pre-warmed to 37 °C. Ejaculates were obtained in the presence of a mare in estrous, using a phantom. Immediately after collection, the gel fraction was removed by filtration through sterile gauze. The volume of the resulting gel-free semen was recorded, and sperm concentration was estimated using SpermaCue (Minitube, Tiefenbach, Germany). Each sample was subsequently diluted in INRA96 extender (Humeco, Huesca, Spain) to achieve a final concentration of 100 × 10^6^ sperm/mL. Both the diluent and the material used during the analyses were kept preheated at 37 °C. For subsequent staining and analysis, three types of preparations were made: unstained smears, eosin–nigrosine–stained smears, and aliquots fixed in 2% glutaraldehyde in 0.33 M cacodylate buffer.

#### 2.2.1. Motility Analysis

Sperm motility analysis was performed using the ISAS PSUS^®^ system (Arquimea Agrotech, Madrid, Spain). A dilution of 20 × 10^6^ sperm/mL was prepared, of which 5 µL were loaded into the specific ISAS motility measurement chambers. Depending on the sample negative phase-contrast was applied. Each field was analyzed in real time (under 1 s), capturing up to 250 consecutive frames at a rate of 200 frames per second.

#### 2.2.2. Viability and Morphological Abnormalities Analysis

For viability and morphology analysis, slides stained with eosin–nigrosin and Giemsa were used as previously described [22]. Briefly, 10 µL of sample at a concentration of 100 × 10^6^ was mixed with 10 µL of eosin–nigrosin solution and placed on a slide for 30 s at 37 °C. Then, samples were stained with Giemsa, previously fixed in 4% formaldehyde in TPB buffer (50 mM Na_2_HPO_4_, 25 mM KH_2_OP_4_, 77 mM K, Na tartrate) in a Coplin jar for 10 min. They were rinsed with running tap water for 10 min, followed by a final rinse with distilled water using a Pasteur pipette. The slides were then stained for 90 min in a Coplin jar containing Giemsa solution, rinsed with distilled water, and air-dried. Finally, the slides were mounted with DPX (BDH, Spain).

The slides were analyzed using a bright field microscope with a 100× objective, employing immersion oil. Viability analysis was performed considering viable spermatozoa as those with pale or unstained post-acrosomal region, indicating intact membranes and exclusion of the vital dye. In contrast, non-viable spermatozoa displayed a darkly stained head (blue/purple) due to loss of membrane integrity and subsequent dye uptake. The Giemsa component provided contrast for the acrosomal borders in both viable and non-viable cells [23]. Morphological abnormalities were assessed by evaluating one hundred spermatozoa, classifying defects in the head, midpiece, and principal piece. Subsequently, the percentage of normal spermatozoa was calculated as one hundred minus the sum of the abnormalities. In addition, a separate count of one hundred spermatozoa was performed to evaluate the presence of cytoplasmic droplets (proximal, medial, or distal). The number of loose heads and spermatozoa with coiled tails was also recorded. The latter parameter was used as an indicator of sample handling in the laboratory.

#### 2.2.3. Acrosome Integrity Analysis

For the acrosome integrity analysis, a Peanut Agglutinin-FITC (PNA-FITC) lectin staining was used [22]. Smears were prepared with 10 μL of sample diluted to 100 × 10^6^ sperm/mL and were previously permeabilized with absolute ethanol 30 s. Subsequently, they were incubated in darkness for 12 min with a solution containing 7.5 µL of PNA-FITC (Sigma-Aldrich, St. Louis, MO, USA) at a final concentration of 15 µg/mL, and 2.5 µL of Hoechst 33258 (concentration 1 mg/mL) (Sigma-Aldrich, St. Louis, MO, USA) in PBS (Gibco).

Samples were examined using a Nikon E50i fluorescence microscope equipped with a triple-band filter (450–490 nm) and a 100× oil immersion objective. For each sample, 100 spermatozoa were evaluated and classified into two categories: spermatozoa with an intact acrosome and spermatozoa with a damaged or lost acrosome. Representative micrographs of staining patterns are provided in Supplementary Figure S1.

#### 2.2.4. Protamination Analysis

For protamine deficiency analysis in spermatozoa, staining with chromomycin A3 fluorochrome (CMA3) was used [22]. Smears were prepared with 10 µL of sample at a concentration of 100 × 10^6^ sperm/mL and allowed to air-dry. The slide was then immersed in Carnoy’s solution (ethanol:glacial acetic acid, 3:1 ratio). Subsequently, 50 µL of a CMA3 solution with final concentration 0.25 mg/mL, was added to the sample and incubated for 20 min at room temperature in the dark. The slides were rinsed with McIlvaine buffer and air-dried, followed by mounting with buffered glycerol.

The samples were then observed under a Nikon E50i fluorescence microscope with a 100× objective using immersion oil with the appropriate filters (460–470 nm). For each sample, 100 spermatozoa were analyzed, distinguishing two staining patterns: spermatozoa with intensely yellow-stained heads were classified as partially or completely protamine-deficient, while spermatozoa with unstained heads were considered to have normal protamine levels. Representative micrographs of both staining patterns are provided in Supplementary Figure S2.

#### 2.2.5. DNA Fragmentation

DNA fragmentation was evaluated using the sperm chromatin structure assay (SCSA) [22]. Semen samples were initially diluted in INRA96 extender to a concentration of 100 × 10^6^ spermatozoa/mL. Immediately after dilution, 100 µL aliquots were snap-frozen in liquid nitrogen. After thawing, samples were further diluted in TNE buffer (0.15 M NaCl, 0.01 M Tris-HCl, 1 mM EDTA; pH 7.4) to reach a final concentration of 2 × 10^6^ spermatozoa/mL. Prepared samples were stored frozen at −20 °C and transported to the School of Agricultural, Forestry and Biotechnology Engineering (University of Castilla-La Mancha, Albacete, Spain) for subsequent analysis. For SCSA processing, 200 µL of each sample were combined with 400 µL of an acid detergent solution (0.08 N HCl, 0.15 M NaCl, 0.1% Triton X-100; pH 1.4) and incubated for 30 s. Immediately thereafter, 1.2 mL of acridine orange staining solution (0.1 M citric acid, 0.126 M Na_2_HPO_4_, 0.0011 M disodium EDTA, 0.15 M NaCl; pH 6.0, 4 °C) was added. Fluorescence signals were measured using a flow cytometer equipped with a 488 nm laser.

DNA fragmentation was expressed as the DNA fragmentation index (DFI), calculated as the proportion of spermatozoa exhibiting red fluorescence relative to the total fluorescence. The DFI cut-off value was determined using a reference sample considered to be free of DNA damage. A DFI histogram was generated from this control, showing a clearly defined main peak corresponding to non-fragmented spermatozoa. Based on this distribution, the threshold for classifying sperm as DNA-damaged was established. In the present study, a DFI value of 23 was set as the upper limit of the non-damaged sperm population.

### 2.3. Statistical Analysis

Prior to statistical analysis, percentage-based parameters were normalized using arcsine transformation, while absolute values were normalized using natural logarithm (ln) transformation. Means, standard errors of the mean (SEM), and data ranges were calculated for all measured parameters, including both blood and semen variables, across the entire study population, regardless of the horse BCS. For each stallion, values obtained from the three ejaculates were averaged to obtain a single representative value per animal, thereby accounting for intra-individual variability. Differences between normo-weight (BCS 4–6) and obese (BCS 7–8) stallions were evaluated using an unpaired *t*-test in GraphPad Prism (version 9.0, GraphPad Software, San Diego, CA, USA), with stallion (*n* = 26) as the experimental unit. Results are presented as mean ± SEM, and statistical significance was defined as *p* < 0.05. As a complementary analysis, stallions at the two extremes of the BCS scale (BCS = 4–5 vs. BCS = 8) were compared using a two-way ANOVA, with BCS group and reproductive parameter as factors, followed by Sidak’s multiple comparisons test; values were likewise averaged per stallion prior to this comparison.

Principal component analyses (PCA) were performed separately for blood and semen parameters to explore patterns of variation and potential correlations among variables. This multivariate technique reduces the dimensionality of the data by transforming the original correlated variables into a smaller set of uncorrelated components (principal components) that capture the maximum variance in the dataset. The first few principal components were examined to identify the variables contributing most to overall variability and to visualize potential clustering or separation within the study population.

## 3. Results

### 3.1. Body Condition, Metabolic and Semen Parameters

#### 3.1.1. Body Condition Score Results

Following assessment of body condition in the entire stallion population, animals were grouped into two categories: 14 stallions exhibited a low BCS with score between 4 and 6, which is considered as a normal condition, and 12 stallions exhibited high BCS with score between 7 and 8, which is regarded as obesity.

#### 3.1.2. Blood Parameters

The results of the analysis of the different endocrinological and metabolic parameters in the stallion’s blood are presented in Table 1. Animals displayed variability in age and body condition score, as well as in metabolic parameters, including lipid profile, glucose, insulin, NEFA, and leptin concentrations.

#### 3.1.3. Semen Traits

The results of seminal parameters analysis are shown in Table 2. Parameters associated with seminal production, such as ejaculate volume and total sperm count, as well as sperm quality parameters, including motility, viability and acrosome integrity, are reported. Additionally, protamination percentage, DNA fragmentation, and sperm maturation status were assessed.

In addition, various sperm morphological characteristics were analyzed, including head abnormalities, the presence of cytoplasmic droplets, and flagellar damage; these results are presented in Table 3.

### 3.2. Comparisons Between Stallions of Different Body Conditions

#### 3.2.1. Comparison of Blood Parameters

The stallion population was divided into two groups according to BCS at the time of blood sampling. Stallions with a BCS between 4 and 6 were assigned to one group, whereas those with a BCS between 7 and 8 were assigned to the second group (Table 4).

Significant differences were observed in parameters related to carbohydrate metabolism. Blood insulin concentration was significantly higher in stallions with higher BCS (*p* = 0.0263), whereas the glucose-to-insulin ratio was significantly higher in stallions with lower BCS (*p* = 0.0376). In addition, significant differences (*p* = 0.0046) were found in triglyceride levels, which were higher in the BCS 7–8 group.

In addition, correlation analyses were performed between blood parameters and BCS. A significant correlation (r = 0.576, *p* = 0.04) was observed between BCS and blood insulin concentration and triglycerides level (r = 0.525, *p* = 0.005). Furthermore, a tendency toward a correlation (r = −0.547, *p* = 0.06) was detected between body condition and NEFA concentrations.

#### 3.2.2. Comparison of Semen Parameters

A comparison of semen parameters was performed between the groups defined in Section 3.2.1 significant differences were observed in the total number of spermatozoa and in total motility (*p* < 0.0001, *p* = 0.0055, respectively) between the two groups, with higher values in horses presenting a BCS of 4 to 6 (Table 5).

### 3.3. Analysis of Groups with Extreme Body Condition Score

Based on the results obtained in the previous sections, an analysis was conducted comparing stallions with extreme BCS values. Stallions with a BCS of 4 and 5 (*n* = 5) were grouped together, while stallions with a BCS of 8 (*n* = 5) formed the second group.

The results obtained from the comparison of blood parameters between both populations are presented in Table 6. Significant differences were observed in triglyceride levels (*p* = 0.0065), with lower concentrations in the normo-weight group compared to the obese group (29.40 ± 3.76 mg/dL vs. 53.20 ± 11.83 mg/dL, respectively). Semen parameters (Table 7) revealed significant differences (*p* < 0.0001) in total number of spermatozoa, with higher values in normo-weight stallions compared to obese stallions (7667.19 ± 471.06 vs. 6369.94 ± 408.52, respectively).

### 3.4. Principal Component Analysis (PCA)

#### 3.4.1. PCA of Blood Parameters

**Figure 1:**
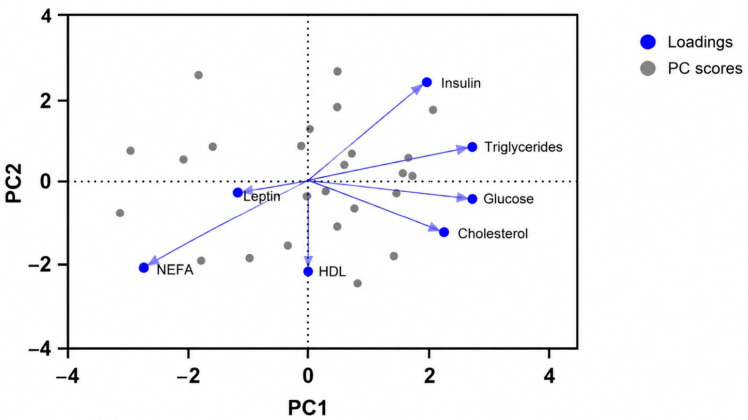
Biplot of the principal component analysis (PCA) of endocrine and metabolic parameters in stallions. Vectors represent the loadings of the variables included in the analysis, and points correspond to individual values. The first principal component (PC1) reflects an axis of obesity and metabolic risk, while the second principal component (PC2) represents variability in lipid metabolism and insulin sensitivity.

Initially, PCA was performed on all analyzed blood parameters (Table 8, Figure 1). The first principal component (PC1: 32.87% of the total variance) represented metabolic risk, because glucose concentration, insulin, triglycerides, and cholesterol showed the highest loadings. The second principal component (PC2: 23.00%) included negative loadings for NEFA concentrations and HDL-cholesterol. The third principal component (PC3: 16.94%) included leptin showing the dominant loading, as did PC4 (13.96%). PC5 and PC6 were not considered highly informative, as no individual variable showed a strong contribution to these components (Table 9).

A Pearson correlation analysis was conducted between BCS and PC1 and PC2 factors. No significant correlations were found (body condition vs PC1, r = 0.3077, *p* = 0.1262; body condition vs PC2, r = 0.2219, *p* = 0.2759).

#### 3.4.2. Principal Component Analysis of Semen Parameters

**Figure 2:**
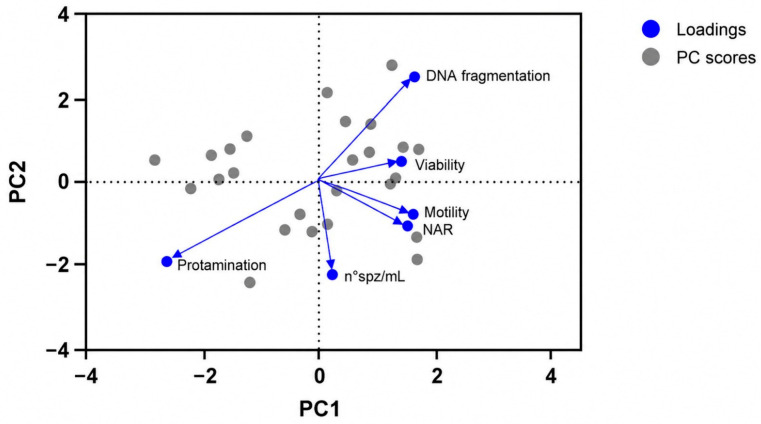
Biplot of the principal component analysis (PCA) of semen parameters in stallions. Vectors represent the loadings of the analyzed semen variables, and points correspond to individual values. The first principal component (PC1) reflects an axis of sperm quality and DNA integrity, while the second principal component (PC2) represents variability in semen production and protamination.

The results of the PCA of the different seminal parameters are presented in Table 10, including eigenvalues, total and cumulative variance (Figure 2). The first principal component (PC1: 35.16% of the total variance) was mainly associated with sperm quality and DNA integrity (Table 11). The second principal component (PC2: 23.98%) primarily reflected semen production variables (total sperm count) and protamination. The third principal component (PC3: 16.99%) was dominated by sperm viability. PC4 and PC5 did not have individual variables showing a strong contribution to these components (Table 11).

Pearson correlation analysis was performed between BCS and PC1 and PC2. The correlation coefficients were r = 0.009 and *p* = 0.9646 for PC1; r = 0.068 and *p* = 0.7406 for PC2, indicating no correlation.

#### 3.4.3. Correlation Between Blood Parameter PCA and Seminal Parameter PCA

**Figure 3:**
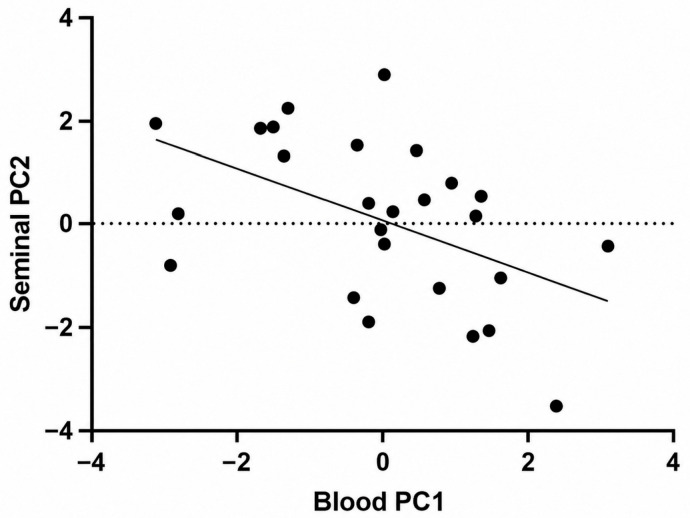
Correlation plot between blood PC1 and seminal PC2. Different points correspond to individual values.

A correlation analysis was conducted between the most representative principal component of each PCA (Table 12). No significant correlation was observed between blood PC1 and seminal PC1; the same was observed for the correlation between blood PC2 and seminal PC1 or PC2. On the other hand, a significant correlation (*p* = 0.012) was found between PC1 of the blood parameters and PC2 of the seminal parameters (Figure 3).

## 4. Discussion

Due to the growing importance of the Purebred Spanish Horse for the European economy [24], the present study investigated obesity-associated alterations in blood and seminal parameters in breeding stallions of this breed. Blood parameters showed considerable variability among stallions. Although a correlation between BCS and circulating insulin levels was identified, not all horses with high BCS exhibited an elevation in this hormone. A similar pattern was observed for seminal parameters, where wide ranges were observed in both high and low BCS groups for parameters such as motility, acrosome integrity, and sperm DNA fragmentation.

In comparisons between stallions with different BCS, significantly higher blood insulin levels, as well as a lower glucose/insulin ratio were observed in horses with higher body condition scores. These findings are consistent with those reported in humans, where obesity is frequently associated with insulin resistance, that is, long-term hyperinsulinemia [25]. Insulin exerts multiple physiological functions, including stimulation of glucose uptake, promotion of lipogenesis, and inhibition of protein degradation. Therefore, obesity is associated with insulin resistance, which in turn plays an important role in the pathogenesis of numerous metabolic disorders [10]. In addition, when comparing populations with extreme body condition scores, higher blood triglyceride concentrations were observed in stallions with higher body condition. The absence of significant differences in leptin concentration between BCS groups was somewhat unexpected, given that leptin is commonly reported as one of the biomarkers most consistently altered in obesity. This finding may be partly explained by the fact that leptin secretion follows a circadian rhythm and is released in a pulsatile manner; as blood samples were collected at a single time point per stallion, this temporal variability may have masked potential differences between groups. Additionally, it has been suggested that plasma leptin concentrations may reflect short-term changes in energy balance rather than chronic body condition status [26], which may further contribute to the lack of a clear association with BCS in the present study.

Regarding the analysis of seminal parameters, significant differences in total sperm count and total motility were observed between the two groups of stallions, which agrees with what has been observed in rodents [27], and in humans [28]. These findings are consistent with the hypothesis that the reproductive alterations observed in stallions with higher body condition scores are associated with metabolic and endocrine changes rather than with a direct effect of increased adipose tissue per se, in line with previous reports [29]. Hyperinsulinemia, among other metabolic alterations, has been proposed as a potential contributing mechanism impairing spermatogenesis [30].

The principal component analysis of blood parameters identified a dominant component (PC1), characterized by high loadings for blood glucose levels, insulin concentrations, and lipid-related parameters, reflecting a metabolic profile potentially relevant to insulin resistance and lipid metabolism. Although no significant correlation was found between BCS and PC1 in the present study, comparable metabolic profiles have been associated with obesity in humans [18] and in other domestic species, such as dogs and cats, in which obesity is associated with dyslipidemia in dogs and with increased insulin resistance in both species [31,32]. Taken together, PC2 and PC3 reflect metabolic variability independent of body condition, suggesting that obesity may not constitute a homogeneous metabolic state across individuals. This concept is supported by previous studies in humans, where severe obesity has not necessarily been associated with the development of metabolic syndrome, as some individuals remain metabolically healthy despite presenting severe obesity [33,34]. In general, results revealed inter-individual differences in the endocrine function of adipose tissue, regardless of the body condition exhibited by the animals.

Conversely, principal component analysis of the seminal parameters revealed that PC1 was dominated by sperm quality and chromatin integrity variables. In the present study, no significant differences were observed between obese and normo-weight individuals with regard to chromatin maturation or direct DNA damage. These findings contrast with those reported by Dupont et al. [35] and Fariello et al. [36], who described higher rates of DNA damage in obese men. However, numerous studies have failed to demonstrate this association, and this issue remains controversial [18], highlighting the need for further research into the effects of obesity on sperm DNA. Regarding acrosome integrity and sperm viability, both parameters are closely dependent on plasma membrane integrity. In fresh semen, membrane stability may be relatively preserved even in the presence of moderate metabolic alterations, and the impact of elevated triglycerides and cholesterol on membrane-dependent parameters may only become apparent under additional stress conditions not present in this study. Regarding DNA fragmentation and protamination, these parameters largely reflect chromatin condensation processes established during earlier stages of spermatogenesis and may therefore be less directly responsive to the current circulating metabolic status. Furthermore, since the metabolic alterations observed in this cohort were moderate in magnitude, the threshold required to induce detectable DNA damage may not have been reached, consistent with the generally low levels of DNA fragmentation and deprotamination observed across the study population. PC2, in turn, was mainly associated with total sperm count and protamination status, the two seminal parameters showing the highest loadings on this component. However, the direct comparison between BCS groups revealed a significantly lower total sperm count in stallions with higher body condition, consistent with other studies concluding that obesity in men is associated with reduced sperm concentration as well as a lower total sperm count [37].

This study presents certain limitations. Although mean age was comparable between BCS groups, the wide age range of the cohort (3–21 years) may have independently influenced individual metabolic parameters, regardless of body condition. In addition, measurements of reproductive hormones such as testosterone, LH, and FSH were not included; the endocrine assessment instead focused on metabolic markers (insulin and leptin), selected on the basis that BCS alone was considered insufficient to characterize the metabolic status of the animals. This choice reflects the primary aim of the study, which was to evaluate seminal quality rather than to provide a comprehensive hormonal profile. Finally, direct fertility outcomes, such as pregnancy or conception rates, were not evaluated; the seminal parameters assessed are recognized as surrogate markers of reproductive potential, although their direct relationship with fertility in this cohort remains to be established.

## 5. Conclusions

Obesity was associated with deleterious endocrine-metabolic alterations, including hyperinsulinemia and dyslipidemia, as well as with reduced total sperm count and total motility. Notably, integration of blood and seminal PCA revealed that increased insulin, triglycerides, and cholesterol were significantly associated with a reduced total sperm count, reinforcing the link between metabolic status and semen quality. These metabolic disturbances potentially compromise the quantity and quality of semen doses prepared for ARTs. Our findings suggest that reproductive changes in stallions with higher body condition scores may be associated with metabolic and endocrine alterations rather than with adiposity per se. It should be noted that causal relationships cannot be established based on the observational design of this study. Further research is warranted to elucidate the underlying mechanisms and to evaluate the long-term impact of these metabolic shifts on overall stallion fertility.

## Figures and Tables

**Table 1:** Age, body condition score (BCS), metabolic and endocrine parameters (mean ± SEM and range, *n* = 26) of Spanish Purebred stallion used in this study.

Parameter	Mean ± SEM	Min–Max Range
Age	11.0 ± 1.24	3–21
BCS	6.34 ± 0.20	4–8
Cholesterol (mg/dL)	105.72 ± 2.33	79.72–132.78
HDL-cholesterol (mg/dL)	55.97 ± 1.14	46–69
Triglycerides (mg/dL)	37.0 ± 3.56	11–100
NEFA (mmol/L)	0.19 ± 0.03	0.051–0.489
Glucose (mg/dL)	85.02 ± 2.17	62.4–101.4
Insulin (µUI/mL)	9.84 ± 1.83	1.81–45.15
Glucose/Insulin	13.61 ± 2.08	2.01–41.93
Leptin (ng/mL)	0.26 ± 0.08	0.01–1.82

**Table 2:** Descriptive statistics of seminal parameters (mean ± SEM and range, *n* = 26).

Parameter	Mean ± SEM	Min–Max Range
Ejaculate volume (mL)	35.44 ± 2.28	5–140
Concentration (×10^6^ sperm/mL)	230.81 ± 8.02	91–407
Total sperm count (×10^6^ sperm)	7217.25 ± 309.31	1010–14,750
Viability (%)	89.30 ± 0.97	45–100
Total motility (%)	82.28 ± 2.42	58.86–92.81
Progressive motility (%)	64.04 ± 3.09	10–95
Normal spermatozoa (%)	52.59 ± 2.19	26–92
Sperm with intact acrosome (%)	72.67 ± 1.55	37–95
Sperm with complete deprotamination (%)	0.42 ± 0.10	0–4
Sperm with partial deprotamination (%)	1.78 ± 0.20	0–8
DNA fragmentation (% tDFI)	13.58 ± 0.56	3.11–25.28
Cellular maturity (% HDS)	7.66 ± 0.32	3.80–17.75

**Table 3:** Morphological characteristics of spermatozoa (mean ± SEM and range, *n* = 26).

Parameter	Mean ± SEM	Min–Max Range
Head abnormalities (%)	5.49 ± 0.39	0–17
Midpiece abnormalities (%)	8.99 ± 0.63	0–28
Principal piece abnormalities (%)	32.91 ± 2.51	0–59
Proximal droplet (%)	12.10 ± 1.04	0–46
Middle droplet (%)	8.21 ± 0.81	0–32
Distal droplet (%)	1.53 ± 0.22	0–9
Coiled spermatozoa (%)	1.58 ± 0.19	0–8
Loose heads (%)	2.60 ± 0.44	0–21

**Table 4:** Comparison of metabolic and endocrine parameters between stallions with different body condition scores (BCS) (mean ± SEM and range). Sample sizes: BCS 4–6, *n* = 14; BCS 7–8, *n* = 12.

Parameter	BCS 4–6		BCS 7–8	
	**mean ± SEM**	**min–max range**	**mean ± SEM**	**min–max range**
Age	10 ± 1.30	5–20	11 ± 1.77	3–21
BCS	5.58 ± 0.14	4–6	7.41 ± 0.12	7–8
Cholesterol (mg/dL)	103.14 ± 3.76	79.72–132.78	109.37 ± 1.97	97.28–120.51
HDL-cholesterol (mg/dL)	55.76 ± 1.33	49–69	56.25 ± 3.75	46–63
Triglycerides (mg/dL)	31.23 ± 2.84 *	11–59	45.17 ± 3.74 *	26–100
NEFA (mmol/L)	0.17 ± 0.04	0.051–0.489	0.20 ± 0.04	0.063–0.479
Glucose (mg/dL)	80.82 ± 2.57	62.4–93.4	90.97 ± 1.63	84.9–101.4
Insulin (µUI/mL)	5.68 ± 0.78 *	1.81–11.63	13.20 ± 3.33 *	4.15–45.15
Glucose/Insulin	18.22 ± 3.19 *	7.54–41.93	10.13 ± 1.71 *	2.02–20.46
Leptin (ng/mL)	0.24 ± 0.28	0.1–0.72	0.28 ± 0.15	0.01–1.82

* Significant differences (*p* < 0.05) between groups.

**Table 5:** Comparison of semen parameters between stallions with different body condition scores (BCS) (mean ± SEM and range). Sample sizes: BCS 4–6, *n* = 14; BCS 7–8, *n* = 12.

Parameter	BCS 4–6		BCS 7–8	
	**mean ± SEM**	**min–max range**	**mean ± SEM**	**min–max range**
Ejaculate volume (mL)	37.15 ± 4.24	10–82	34.20 ± 4.04	5–85
Concentration (×10^6^ sperm/mL)	219.45 ± 12.88	102–407	221.17 ± 9.42	100–359
Total sperm count (×10^6^ sperm)	8311.27 ± 649.83 *	6598–9694	6054.67 ± 343.09 *	5310–6824
Viability (%)	88.78 ± 1.46	80.5–99	90.63 ± 1.78	81–95.33
Total motility (%)	87.96 ± 1.23 *	73.75–97.65	80.57 ± 2.21 *	51.37–93.96
Progressive motility (%)	70.41 ± 11.27	20–90	68.02 ± 9.86	30–90
Normal sperm (%)	60.63 ± 4.06	40–80	54.93 ± 4.09	43–83
Sperm with intact acrosome (%)	71.81 ± 3.04	53–89.67	73.94 ± 2.83	54.5–85.33
Sperm with total deprotamination (%)	1.95 ± 0.67	0–5	1.85 ± 0.75	0–4
Sperm with partial deprotamination (%)	0.60 ± 0.40	0–3	1.20 ± 0.58	0–4
DNA fragmentation (% tDFI)	13.25 ± 0.96	3.33–35.65	13.91 ± 1.19	3.11–22.63
Cellular maturity (% HDS)	7.10 ± 0.57	6.33–12.66	7.74 ± 0.76	3.83–13.80

* Significant differences (*p* < 0.05) between groups.

**Table 6:** Comparison of metabolic and endocrine parameters between stallions with extreme body condition scores (BCS) (mean ± SEM and range). Sample sizes: BCS 4–5, *n* = 5; BCS 8, *n* = 5.

Parameter	BCS 4–5		BCS 8	
	**mean ± SEM**	**min–max range**	**mean ± SEM**	**min–max range**
Age	10.2 ± 1.35	6–14	12.4 ± 2.97	6–21
Cholesterol (mg/dL)	105.98 ± 7.36	84.47–123.3	111.22 ± 4.23	97.28–120.51
HDL-cholesterol (mg/dL)	56.60 ± 3.41	49–69	55.40 ± 2.25	51–63
Triglycerides (mg/dL)	29.40 ± 3.76 *	22–40	53.20 ± 11.83 *	35–100
NEFA (mmol/L)	0.23 ± 0.07	0.08–0.49	0.12 ± 0.03	0.06–0.20
Glucose (mg/dL)	82.60 ± 2.78	75.9–89.4	94.38 ± 2.45	88.1–101.4
Insulin (µUI/mL)	8.22 ± 3.75	1.81–21.95	19.43 ± 6.81	7.9–45.15
Glucose/Insulin	20.77 ± 6.83	3.52–25.65	7.23 ± 1.98	2.01–12.35
Leptin (ng/mL)	0.11 ± 0.04	0.02–0.24	0.43 ± 0.35	0.01–1.82

* Significant differences (*p* < 0.05) between groups.

**Table 7:** Comparison of semen parameters between stallions with extreme body condition scores (BCS) (mean ± SEM and range). Sample sizes: BCS 4–5, *n* = 5; BCS 8, *n* = 5.

Parameter	BCS 4–5		BCS 8	
	**mean ± SEM**	**min–max range**	**mean ± SEM**	**min–max range**
Ejaculate volume (mL)	53.13 ± 4.87	39.33–69.67	28.93 ± 3.74	19.33–40.0
Concentration (×10^6^ sperm/mL)	168.60 ± 17.46	130–226	260.40 ± 20.13	204–330
Total sperm count (×10^6^ sperm)	7667.19 ± 471.06 *	6914–9694	6369.94 ± 408.52 *	5153–6824
Viability (%)	91.20 ± 2.67	83–99	86.40 ± 3.45	77–95
Total motility (%)	89.37 ± 1.56	83.58–94.92	85.74 ± 1.48	71.46–92.61
Progressive motility (%)	70.42 ± 11.27	40–88	68.02 ± 9.86	36–90
Normal sperm (%)	97.73 ± 0.19	95–100	98 ± 0.39	48–85
Sperm with intact acrosome (%)	70.53 ± 5.99	52–86	70.33 ± 6.25	48–85
Sperm with total deprotamination (%)	0.52 ± 0.11	0–6	0.55 ± 0.12	0–6
Sperm with partial deprotamination (%)	1.69 ± 0.16	0–6	1.66 ± 0.18	0–7
DNA fragmentation (% tDFI)	11.02 ± 2.28	5.93–17.50	12.13 ± 2.69	4.86–18.02
Cellular maturity (% HDS)	5.26 ± 0.64	4.54–6.53	7.01 ± 2.32	3.83–11.54

* Significant differences (*p* < 0.05) between groups.

**Table 8:** PCA of blood parameters: eigenvalues, individual variance, and cumulative variance.

PC Factor	Eigenvalues	Total Variance (%)	Cumulative Variance (%)
PC1	2.30	32.87	32.87
PC2	1.61	23.00	55.87
PC3	1.19	16.94	72.80
PC4	0.97	13.96	86.76
PC5	0.51	7.22	96.98
PC6	0.26	3.70	97.68

**Table 9:** Variable loadings of metabolic parameters and their contributions to each principal component (PC).

Parameters	PC1	PC2	PC3	PC4	PC5	PC6
Cholesterol	0.683	−0.465	0.157	−0.356	−0.292	−0.257
HDL_cholesterol	0.005	−0.781	0.468	−0.271	0.109	0.288
Triglycerides	0.845	0.127	−0.106	0.380	−0.082	0.259
NEFA	−0.167	−0.741	−0.366	0.365	0.352	−0.138
Glucose	0.865	−0.185	−0.328	0.147	0.025	−0.019
Insulin	0.582	0.420	0.430	−0.163	0.508	−0.102
Leptin	−0.071	−0.088	0.708	0.671	−0.138	−0.113

**Table 10:** PCA of semen parameters: eigenvalues, individual variance, and cumulative variance.

PC Factor	Eigenvalues	Total Variance (%)	Cumulative Variance (%)
PC1	2.11	35.16	35.16
PC2	1.44	23.98	59.14
PC3	1.02	16.99	76.23
PC4	0.70	11.69	87.82
PC5	0.47	7.79	95.61

**Table 11:** Variable loadings of seminal parameters and their contributions to each principal component (PC).

Parameters	PC1	PC2	PC3	PC4	PC5
Total sperm count	0.116	−0.749	0.073	−0.647	−0.011
Total motility	0.788	−0.316	0.180	0.242	−0.335
Sperm with intact acrosome	0.724	−0.391	0.208	0.274	0.409
Viability	0.458	0.059	−0.842	−0.081	0.206
Protamination	−0.398	−0.654	−0.459	0.321	−0.244
DNA fragmentation	0.761	0.436	−0.129	−0.195	−0.291

**Table 12:** Pearson’s correlation coefficients (r) and corresponding *p*-values among PCA.

	PC1 (Seminal Parameters)		PC2 (Seminal Parameters)	
	*r*	*P*	*r*	*P*
**PC1** **(Metabolic parameters)**	0.259	0.201	−0.483	0.012 *
**PC2** **(Metabolic parameters)**	0.016	0.937	0.107	0.601

* Statistically significant correlations (*p* < 0.05).

## Data Availability

The data presented in this study are available on request from the corresponding author due to privacy and confidentiality restrictions regarding the animals’ owners.

## Supplementary Materials

The following supporting information can be downloaded at https://www.mdpi.com/article/10.3390/ani16152347/s1: Figure S1: Purebred Spanish Horses spermatozoa staining with PNA-FITC for the assessment of acrosome integrity; Figure S2: Purebred Spanish Horses spermatozoa staining with Chromomycin A3 for the assessment of protamination.

## Abbreviations

The following abbreviations are used in this manuscript:

ANOVA	Analysis of variance
BCS	Body Condition Score
CMA3	Chromomycin A3
DFI	DNA fragmentation index
HDL-C	High-density lipoprotein cholesterol
NEFA	Non-esterified fatty acid
PCA	Principal Component Analysis
PNA	Peanut Agglutinin
SCSA	Sperm Chromatin Structure Assay
SEM	Standard Errors of the Mean
