# Supplementary material for: Body Condition, Metabolic Parameters and Semen Traits in Purebred Spanish Horses
Source: Animals (Basel). 2026 Aug 1;16(15):2347. doi: 10.3390/ani16152347 (PMC13463500; doi:10.3390/ani16152347)
Supplement: Supplementary file 1 [file animals-16-02347-s001.zip › animals-4470403-supplementary.pdf]

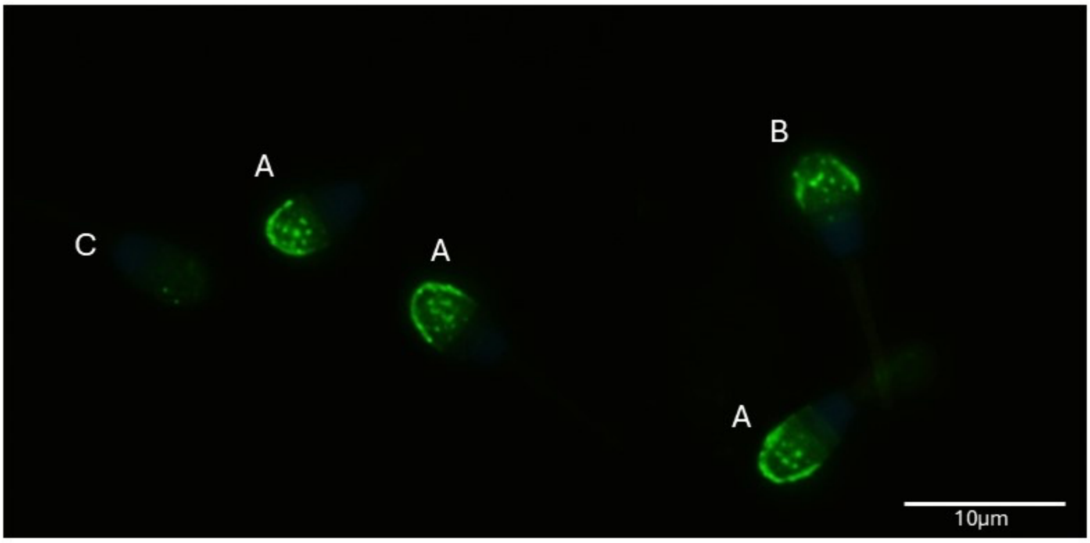

Figure S1. Purebred Spanish Horses spermatozoa staining with PNA-FITC for the assessment of acrosome integrity. A: intact acrosom (NAR) with uniform green fluorescence. B: Damaged acrosome (DAR) with non-uniform fluorescence. C: No acrosome (MAR) without fluorescence.

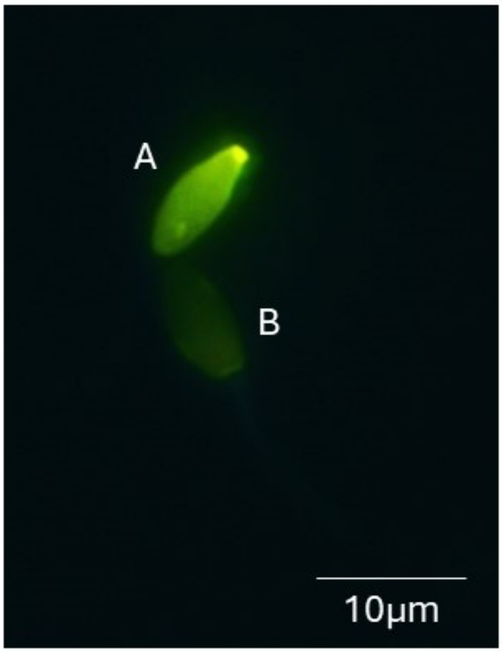

Figure S2. Purebred Spanish Horses spermatozoa staining with Chromomycin A3 for the assessment of protamination. A: sperm completely deprotaminated with intesive stained. B: sperm with normal protamin with light stained.
